# The multi-strategy hybrid forecasting base on SSA-VMD-WST for complex system

**DOI:** 10.1371/journal.pone.0300142

**Published:** 2024-04-18

**Authors:** Huiqiang Su, Shaojuan Ma, Xinyi Xu

**Affiliations:** 1 School of Mathematics and Information Science, North Minzu University, Yinchuan, China; 2 Ningxia Key Laboratory of Intelligent Information and Big Data Processing, Yinchuan, China; Buckinghamshire New University - High Wycombe Campus: Buckinghamshire New University, UNITED KINGDOM

## Abstract

In view of the strong randomness and non-stationarity of complex system, this study suggests a hybrid multi-strategy prediction technique based on optimized hybrid denoising and deep learning. Firstly, the Sparrow search algorithm (SSA) is used to optimize Variational mode decomposition (VMD) which can decompose the original signal into several Intrinsic mode functions (IMF). Secondly, calculating the Pearson correlation coefficient (PCC) between each IMF component and the original signal, the subsequences with low correlation are eliminated, and the remaining subsequence are denoised by Wavelet soft threshold (WST) method to obtain effective signals. Thirdly, on the basis of the above data noise reduction and reconstruction, our proposal combines Convolutional neural network (CNN) and Bidirectional short-term memory (BiLSTM) model, which is used to analyze the evolution trend of real time sequence data. Finally, we applied the CNN-BiLSTM-SSA-VMD-WST to predict the real time sequence data together with the other methods in order to prove it’s effectiveness. The results show that SNR and CC of the SSA-VMD-WST are the largest (the values are 20.2383 and 0.9342). The performance of the CNN-BiLSTM-SSA-VMD-WST are the best, MAE and RMSE are the smallest (which are 0.150 and 0.188), the goodness of fit *R*^2^ is the highest(its value is 0.9364). In contrast with other methods, CNN-BiLSTM-SSA-VMD-WST method is more suitable for denoising and prediction of real time series data than the traditional and singular deep learning methods. The proposed method may provide a reliable way for related prediction in various industries.

## 1 Introduction

With the noise influence of complex time series data, the accuracy of prediction in many industries can not reach the ideal effect. To address the strong noise problem of complex time series [1, 2], traditional denoising methods have already been used widely, such as Wavelet analysis [3], Singular spectrum analysis [4] and Fourier transform [5]. Nguyen et al. [3] used wavelet analysis to reduce the noise of natural gas price time series and input the reduction sequence into the prediction model to achieve higher prediction accuracy. Wen et al. [4] applied Singular spectrum analysis to decompose stock time series and predicted the trend based on Support vector machine.

In relation to the traditional methods, empirical mode decomposition(EMD) has obvious advantages in processing complex time series data [6], which can decompose complex time series into signal components from high frequency to low frequency. However, sometimes EMD would suffer from the mode aliasing and end effect problems [7, 8]. Wu and Huang introduced the ensemble empirical mode decomposition(EEMD) as a solution to the drawbacks of EMD [9], in which adaptive sequential diagnostic algorithm combined with EEMD was used to improve the stability and generalization capacity of the prediction model with lower error. However, a lot of redundant information was generated. To address the problems mentioned above, Drag-omiretskiy et al. [10] presented variational mode decomposition(VMD) which can separate signals into their modal components at various frequencies. VMD method can effectively solve EMD mode aliases and end effects, which has stronger robustness [11, 12]. Nevertheless, the parameters in VMD are indeterminate and different parameter settings will give different results. Therefore, finding the best solution for the parameters in VMD is currently a significant problem [13]. Yang et al. [14] employed a genetic algorithm to maximize the kurtosis of the index set as the objective function in order to estimate the VMD parameters in an adaptive manner. Yan and co-author [15] utilized the cuckoo search algorithm to acquire VMD parameters with the minimal mean envelope entropy as the optimization aim. Zhou et al. [16] utilized the particle swarm optimization approach and the average permutation entropy as the fitness function to optimize. While the previous techniques have yielded some satisfactory outcomes, they were all designed with a single assessment index as the goal function to be optimized rather than a more comprehensive global optimization. To deal with the uncertain effects from collecting historical data, many scholars used the wavelet transform (WT) [17–19] which can remove random fluctuations for prediction. Artificial neural network was utilized to predict wind power based on WT [20]. A hybrid model based on Discrete wavelet threshold was presented to prediction [21]. He et al. [22] successfully predicted monthly precipitation and climatic indices using a hybrid wavelet neural network model. In fact the single denoising methods can’t eliminate noise well [23, 24] for the complex time series data, so scholars began to pay attention to hybrid denoising methods. Chao et al. [25] used TVF-EMD-ENN to predict precipitation time series data, which achieved higher prediction accuracy than EMD. Lv et al. [26] choosed WT and Singular value decomposition effectively to suppress multi-source noise. Lin et al. [27] applied EMD and WT to process the noise signal. Dao et al. [28] used WT and EEMD to complete the effective removal of uncertain noise in fault signals. Song et al. [29] choosed the principle of multiple wavelet denoising to denoise NMR spectra, which showed that the hybrid method effectively to removes a series of spikes comparing with WT method.

Based on above analysis, the VMD parameter optimization model with comprehensive evaluation index is proposed in this paper, which can obtain the optimal VMD parameters in the global scope. Additionally, in order to overcome the noise redundancy problems for single denoising method, a novel time series denoising strategy is presented in this paper based on the hybrid optimized VMD and wavelet soft threshold(WST) to accurate trend evolution. This paper is presented as follows. In section 2, we build a model for optimizing VMD with SSA by establishing a new comprehensive fitness function. In section 3, a hybrid noise reduction model is given through PCC. Finally, we verify the theoretical model with real time series data by numerical experiment, CNN-BiLSTM-SSA-VMD-WST method is proved more suitable for denoising and prediction of real time series data than the traditional and single deep learning methods.

## 2 Correlation theory

### 2.1 Variational mode decomposition

VMD is used to decompress the original signal into IMF components and find the best variational mode solution, which is a completely non-recursive model [30].

For the optimal solution of the constrained variational model, the principle is as follows [30]:

The mode equation is explained as an amplitude frequency signal, which is defined as follows Eq (1):

$$\begin{array}{c} s_{k}(t)=a_{k}(t)\;\text{cos}\;[\phi (t)] \end{array}$$ (1)

where *a*_*k*_(*t*) is corresponding vibration amplitude of the IMF, the derivative of *ϕ*(*t*) is the frequency of the IMF. The variational solution principle of VMD is as follows:

1. The analytic signal corresponding to each *s*_*k*_(*t*) is calculated using Hilbert transform to get frequency of single-sided defined as follows Eq (2):
  $$\begin{array}{c} \left(\delta \left(t\right)+\frac{j}{\pi t}\right)*s_{k}(t) \end{array}$$ (2)
  where *δ*(*t*) is the impact function, and its expression is as follows Eq (3):
  $$\begin{array}{c} \delta \left(t\right)=\{\begin{array}{cc} \infty & t=0\\ 0 & t\neq 0 \end{array} \end{array}$$ (3)
2. Add the *e*^−*jw*_*k*_*t*^ and pass the spectrum of each modal component to the corresponding baseband is defined as follows Eq (4):
  $$\begin{array}{c} {}[\left(\delta \left(t\right)+\frac{j}{\pi t}\right)*s_{k}(t)]\left(e^{-jw_{k}t}\right) \end{array}$$ (4)
3. The principle of VMD can be rewritten as an optimization problem with constraints as follows Eq (5):
  $$\begin{array}{c} \underset{\left\{s_{k}\}\{w_{k}\right\}}{\text{min}}\;\{\sum_{k=1}^{K}{\left\|\partial_{t}[\left(\delta \left(t\right)+\frac{j}{\pi t}\right)*s_{k}(t)]\left(e^{-jw_{k}t}\right)\right\|}_{2}^{2}\},s.t\sum_{k}s_{k}\left(t\right)=f\left(t\right) \end{array}$$ (5)
  where *s*_*k*_(*t*) is show in Eq (1), *w*_*k*_ is the frequency center of every modal component, K is the number of iterations, *f*(*t*) is the original signal, *s*.*t* is the bound term, * is the convolution calculation symbol.
4. Add a penalty factor *α* and an improved Lagrange formula to solve Eq (5), convert ordinary variation problems into unconstrained variation problems. An extended Lagrange expression is obtained as follows Eq (6):
  $$\begin{array}{l} L\left(\left\{s_{k}\right\},\left\{w_{k}\right\},\left\{\lambda \right\}\right)=\alpha \sum_{k=1}^{K}{\left\|\partial_{t}\left[(\delta (t)+\frac{j}{\pi t})*s_{k}\left(t\right)\right](e^{-jw_{k}t})\right\|}_{2}^{2}\\ +{\left\|f(t)-\sum_{k=1}^{K}s_{k}(t)\right\|}_{2}^{2}+\langle \lambda (t),f(t)-\sum_{k=1}^{K}s_{k}(t)\rangle \end{array}$$ (6)
  where *α* is the second penalty factor which can be adjusted to achieve the completeness of the VMD.
5. After processing, the optimal solution expression of the constrained variational model is obtained as follows Eq (8):
  $$\begin{array}{c} \hat{s_{k}^{n+1}}\left(w\right)=\frac{\hat{f}\left(w\right)-\sum_{i\neq k}{\hat{s}}_{t}\left(w\right)+\frac{\lambda (w)}{2}}{1+2\alpha {\left(w-w_{k}\right)}^{2}} \end{array}$$ (7)
  $$\begin{array}{c} w_{k}^{n+1}=\frac{\int_{0}^{\infty }{\left|{\hat{s}}_{k}\left(w\right)\right|}^{2}dw}{\int_{0}^{\infty }{\left|s_{k}\left(w\right)\right|}^{2}dw} \end{array}$$ (8)

### 2.2 SSA algorithm for parameter optimization based on VMD

There are two important parameters that need to be set in the VMD, namely the number of signal decomposition *K* and the penalty parameter *α*. If the *K* is set to a small value, it will lead to insufficient signal decomposition; If the *K* is greater than the number of useful component signals contained in the analyzed signal, it will cause the analyzed signal to be over decomposed. In addition, the penalty parameter *α* in the VMD is used to ensure the accuracy of signal reconstruction. In this paper, the parameter optimization of VMD based on SSA algorithm is constructed, which can get the optimized parameters quickly and accurately. SSA was first proposed by Xue [31] in 2020. This algorithm imitates the foraging behavior of the sparrow population for target optimization, and has strong optimization ability and fast convergence speed.

The original signal is decomposed into *K* modal components by VMD. If the modal component contains less noise, the information related to the signal of the original time series data will be more obvious and the sample entropy will be smaller. The ratio of the sample entropy and PCC is used as a comprehensive evaluation index to construct objective function. In this paper, the procedure for optimizing VMD parameters is transformed into seek the ratio of minimum sample entropy and PCC by SSA.

Sample entropy can measure the irregularity and complexity of the signals. The lower the entropy, the stronger the regularity of information. PCC is a good measure for the correlation between two data sets shown as follows Eq (9):

$$\begin{array}{c} SampEn\left(m,r,N\right)=-ln[\frac{B^{m+1}\left(r\right)}{B^{m}\left(r\right)}] \end{array}$$ (9)

where *N* is the sequence length, *B*^*m*^(*r*) is the probability of matching *m* point before and after signal reconstruction under tolerance *r*. The value of Eq (9) is related to the selection of *m*, *r*, *N*. Different embedding dimensions *m* and similar tolerances *r* correspond to different sample entropy. In general, *m*=1 or 2, *r*=0.1∼0.25*SD*_*x*_, *SD*_*x*_ is the standard deviation of the new sequence after decomposition. In this paper, we choose *m*=2, *r*=0.25*SD*_*x*_. *R* is the degree of correlation between the subsequence and the original sequence is defined as follows Eq (10),

$$\begin{array}{c} R=\frac{cov(X,Y)}{\sigma_{X}\sigma_{Y}} \end{array}$$ (10)

where *X* and *Y* are the sequences before and after reconstruction. *Fit* is fitness function is defined as follows Eq (11),

$$\begin{array}{c} Fit=(\frac{SampEn(m,r,N)}{R})\cdot D \end{array}$$ (11)

where *D* = *log* 10(*d*), *d* is the number of estimated center frequencies for the modes.

**Fig 1:**
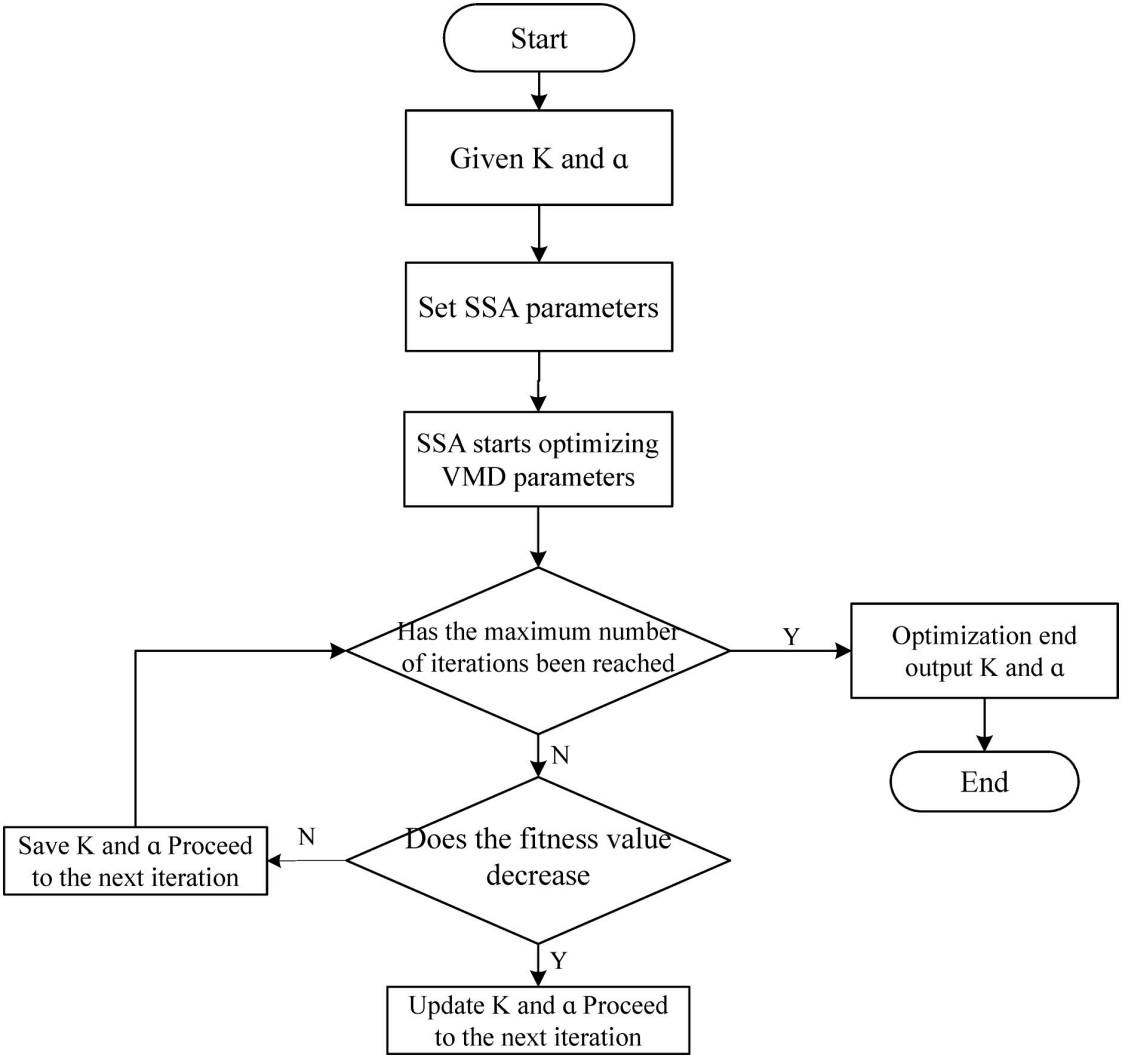
Flowchart of VMD optimization in SSA.

The minimum of *Fit* means that sample entropy is minimum and *R* is maximum. At the moment, the new sequence signal is simpler. We can make decomposition layers *K* ∈ [2, 15] and quadratic penalty factor *α* ∈ [200, 4000] [32]. The following Fig 1 shows the detailed procedure for SSA-VMD.

### 2.3 Wavelet Soft threshold denoising

**Fig 2:**
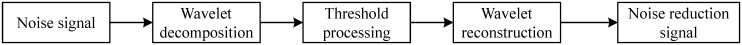
Flow of wavelet threshold denoising.

Donoho [17, 18] proposed the wavelet threshold denoising, reducing irrelevant signals in the original signal is its basic idea. The process of wavelet threshold denoising is shown in Fig 2.

In the two processes of wavelet decomposition and reconstruction, the different types of wavelet bases (dbN, symN, coifN, N is an integer) directly affect signal decomposition and reconstruction. Generally, the number of decomposition layers is chosen based on the properties of the signal. The choice of wavelet threshold value and threshold function also affects the denoising impact. There are four kinds of wavelet threshold value: heuristic threshold, fixed threshold, unbiased risk estimation threshold and minimax threshold. The unbiased risk threshold is more suitable for situations where the noise is similar to additive white Gaussian noise. Minimax threshold is more suitable for signal filtering with low signal-to-noise ratio (SNR) [33]. The heuristic threshold is the synthesis of unbiased risk estimation threshold and fixed threshold. If the threshold value is too small, the denoising effect will be unsatisfactory. If the threshold value is too large, some effective signals will be eliminated. The principle of threshold is as follows [27].

The fixed threshold which is shown as follows Eq (12):

$$\begin{array}{c} \lambda =2\sqrt{lnN} \end{array}$$ (12)

where *N* is the original signal length. Standard deviation of the noise is defined as follows Eq (13):

$$\begin{array}{c} \sigma =\frac{median|w_{j,k}|}{0.6745} \end{array}$$ (13)

where *w*_*j*,*k*_ is the original wavelet coefficient.

The noise reduction effect and threshold function selection are closely related. Different threshold functions have different noise reduction effects, mainly including hard threshold function and soft threshold function. Hard threshold function is presented as follows Eq (14):

$$\begin{array}{c} \eta \left(w_{j,k},\lambda \right)=\{\begin{array}{cc} w_{j,k} & |w_{j,k}|\geq \lambda \\ 0 & |w_{j,k}|<\lambda \end{array} \end{array}$$ (14)

Soft threshold function is shown as Eq (15)

$$\begin{array}{c} \eta \left(w_{j,k},\lambda \right)=\{\begin{array}{cc} sgn\left(w_{j,k}\right)\left(|w_{j,k}|-\lambda \right) & |w_{j,k}|\geq \lambda \\ 0 & |w_{j,k}|<\lambda \end{array} \end{array}$$ (15)

where *sgn*(⋅) is a symbolic function, and the expression is shown as Eq (16):

$$\begin{array}{c} sgn\left(t\right)=\{\begin{array}{cc} 1 & t>0\\ -1 & t<0 \end{array} \end{array}$$ (16)

According to Eqs (14) and (15), the hard threshold function can eliminate the wavelet coefficient which lower than the threshold value in the space. The soft threshold function is to further improve the residence of the hard threshold value, and the signal with the soft threshold value has better smoothness after denoising. Therefore, this paper selects the soft threshold function to denoise the time series signal.

### 2.4 CNN-BiLSTM model

#### 2.4.1 Convolutional neural network

CNN for time series prediction have been paid more attention recently. Three components typically make up CNN: the convolution layer, the pooling layer, and the fully connected layer [34]. In this study, after the experimental data is input into CNN, the convolution layer analyzes the input experimental data using various convolutions, and then the activation function endow nonlinear features for the experimental data. As a result, the experimental data gets the local feature information and achieves the purpose of extracting features. Subsequently, the pooling layer will reduce dimensionality sampling and connect more important data from the convolutional output for making the output feature components more stable. Finally, the fully connected layer creates column vectors of a specific length from the features output from the pooling layer and transmits to the Bidirectional long short-term memory (BiLSTM) network for subsequent operations.

#### 2.4.2 Bidirectional Long Short-Term Memory network

**Fig 3:**
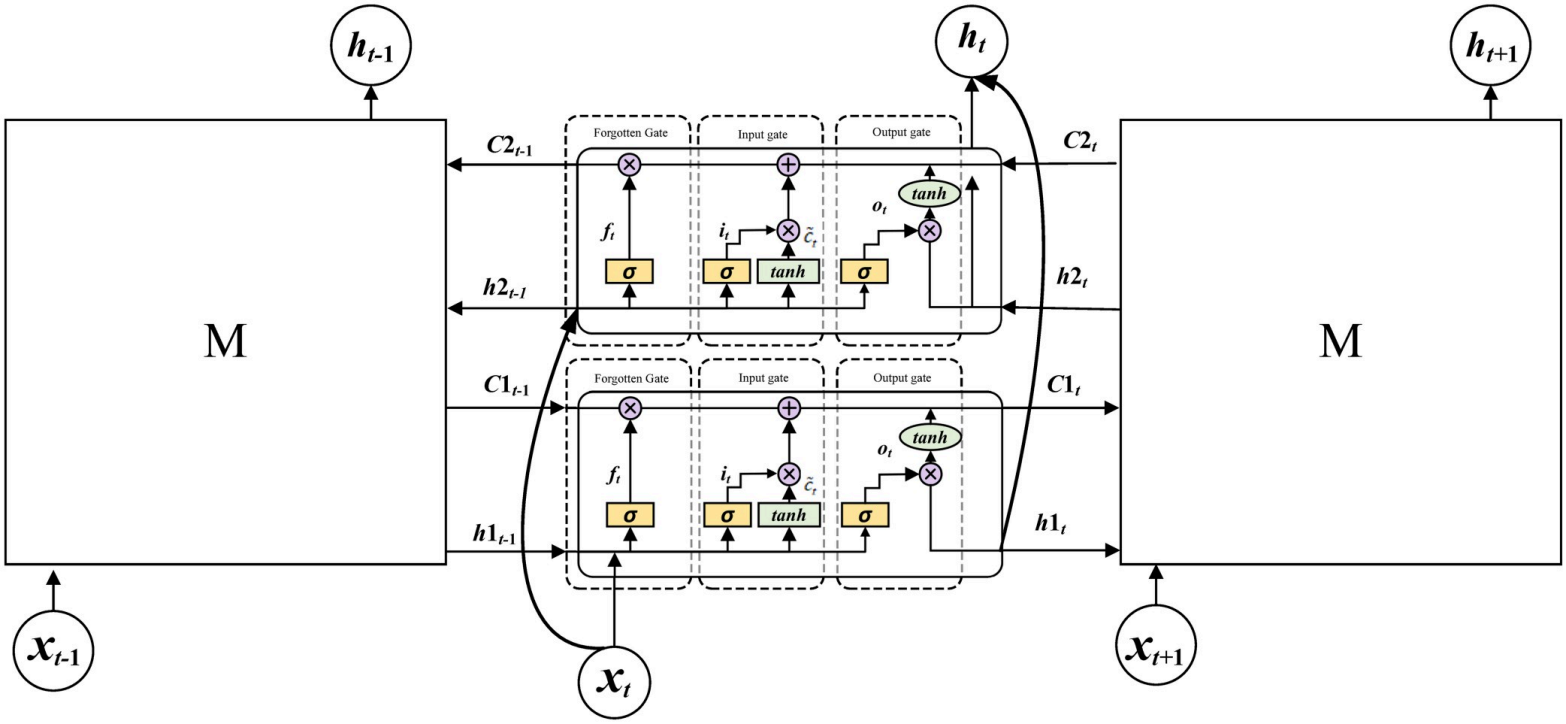
Network structure of BiLSTM.

BiLSTM is able to further processed the results of the CNN, and the time features of experimental data are extracted with the memory function. BiLSTM is developed from the Recurrent neural network (RNN) [35], which can enter data into the network for calculation at every time point, and every hidden layer sends its output immediately to the following time point and the next layer of the network. Its structure is shown in Fig 3.

Each hidden layer consists of two Long short term memory (LSTM), a forward LSTM can obtain the output data from the previous moment and compute it for the following moment. A backward LSTM can obtain the output data from the following moment and compute it for the previous moment. There are four gated units in each LSTM computing unit, they are input gate (*i*_*t*_), output gate(*o*_*t*_), control gate(*c*_*t*_)and forget gate(*f*_*t*_), and M is the network module. The relationship between the various gates are as follow [34, 35]:

The forget gate determines how much the input information *h*_*t*−1_of the previous moment is stored in the current control gate(*c*_*t*_), the relationship between the output *f*_*t*_ and the current time input *x*_*t*_ is shown as follows Eq (17):

$$\begin{array}{c} f_{t}=\sigma (w_{f}\cdot [h_{t-1},x_{t}+b_{f}]) \end{array}$$ (17)

where *w*_*f*_ and *b*_*f*_ are the weights and biases entered for the forget gate respectively, *σ* is the sigmoid activation function that gives the nonlinear properties of the network.

How much of the input data is currently reserved for *c*_*t*_ is determined by the input gate. The intermediate variable ${\tilde{c}}_{t}$ is used as confirmed whether the cell state *i*_*t*_ will be added. The related functional connection is shown as follows Eqs (18) and (19):

$$\begin{array}{c} {\tilde{c}}_{t}=\text{tanh}\;(w_{c}\cdot [h_{t-1},x_{t}]+b_{c}) \end{array}$$ (18)

$$\begin{array}{c} i_{t}=\sigma (w_{i}\cdot [h_{t-1},x_{t}+b_{i}]) \end{array}$$ (19)

where *w*_*c*_ and *w*_*i*_ are the middle weight parameters variables ${\tilde{c}}_{t}$, *i*_*t*_ respectively. *b*_*c*_ and *b*_*i*_ are the intermediate variables ${\tilde{c}}_{t}$ respectively, *i*_*t*_ is the bias parameters, tanh is the activation function.

The amount that the current control gate *c*_*t*_ is transferred to the current time output *h*_*t*_ is determined by the output gate. The related functional connection is shown as follows Eq (22):

$$\begin{array}{c} c_{t}=c_{t-1}\cdot f_{t}+i_{t}\cdot {\tilde{c}}_{t} \end{array}$$ (20)

$$\begin{array}{c} o_{t}=\sigma (w_{o}\cdot [h_{t-1},x_{t}+b_{o}]) \end{array}$$ (21)

$$\begin{array}{c} h_{t}=o_{t}\cdot \text{tanh}\left(c_{t}\right) \end{array}$$ (22)

where *w*_*o*_ and *b*_*o*_ are the weight parameters and bias parameters of the output gate respectively, *c*_*t*_ is the outcome of control gate. The result of the LSTM computation unit is denoted by *h*_*t*_.

The sum of the output values from the forward and backward LSTM units at time *t* is the overall output value of the BiLSTM computing unit. The particular formula for calculating is as follows Eq (25):

$$\begin{array}{c} \vec{h_{t}}=LSTM\left(h_{t-1},x_{t},c_{t-1}\right) \end{array}$$ (23)

$$\begin{array}{c} \overset{\leftarrow }{h_{t}}=LSTM\left(h_{t+1},x_{t},c_{t+1}\right) \end{array}$$ (24)

$$\begin{array}{c} h_{t}=\vec{h_{t}}⊙\overset{\leftarrow }{h_{t}} \end{array}$$ (25)

where ⊙ represents the concatenation of two vectors.

#### 2.4.3 CNN-BiLSTM model

**Fig 4:**
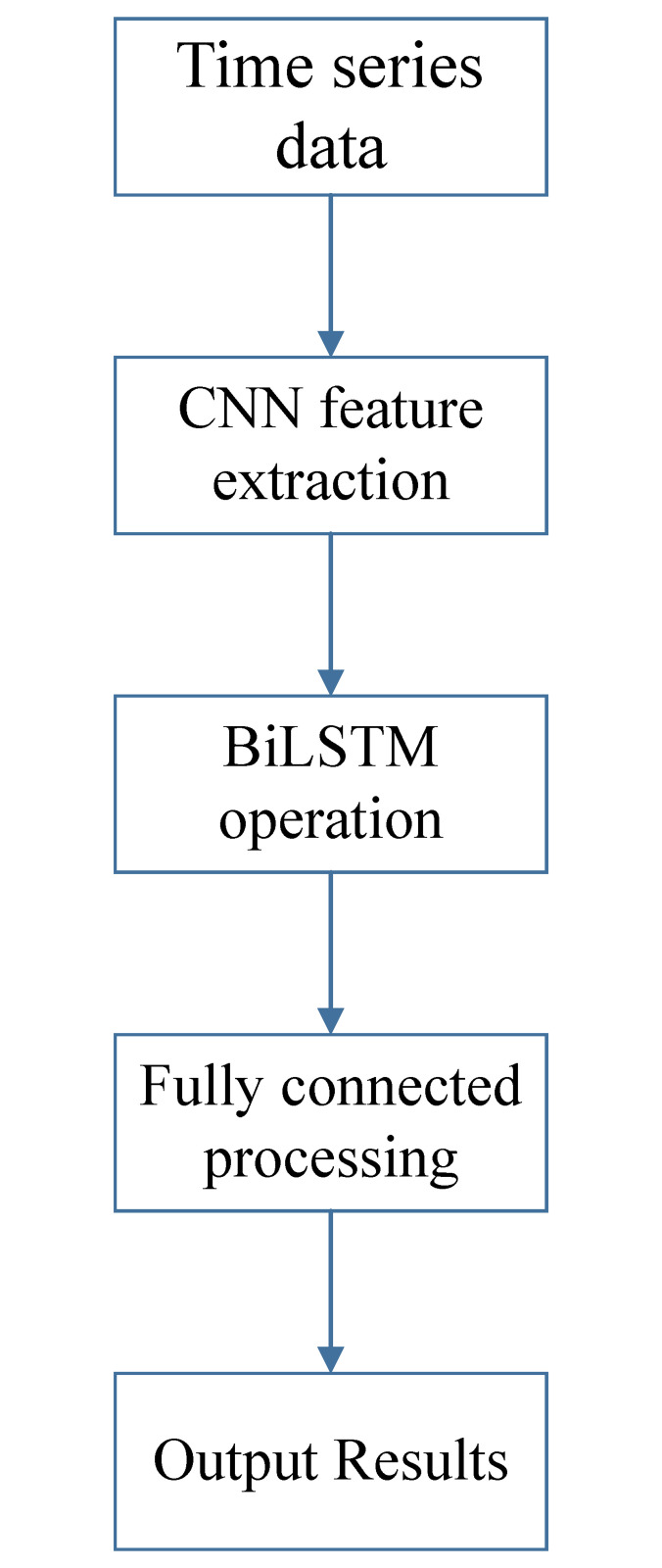
Operation procedure of CNN-BiLSTM model.

CNN-BiLSTM has not only the advantages of CNN, but also the strong information memory ability of BiLSTM. In this paper, CNN is first used for convolution processing of the input time series data, and then BiLSTM is extracted the features of the time series data, which retains the original information of the data to a large extent. Finally, the results are output from the fully connected layer, which significantly raises prediction accuracy. The process is shown in Fig 4.

## 3 Hybrid denoising strategy for SSA-VMD-WST

**Fig 5:**
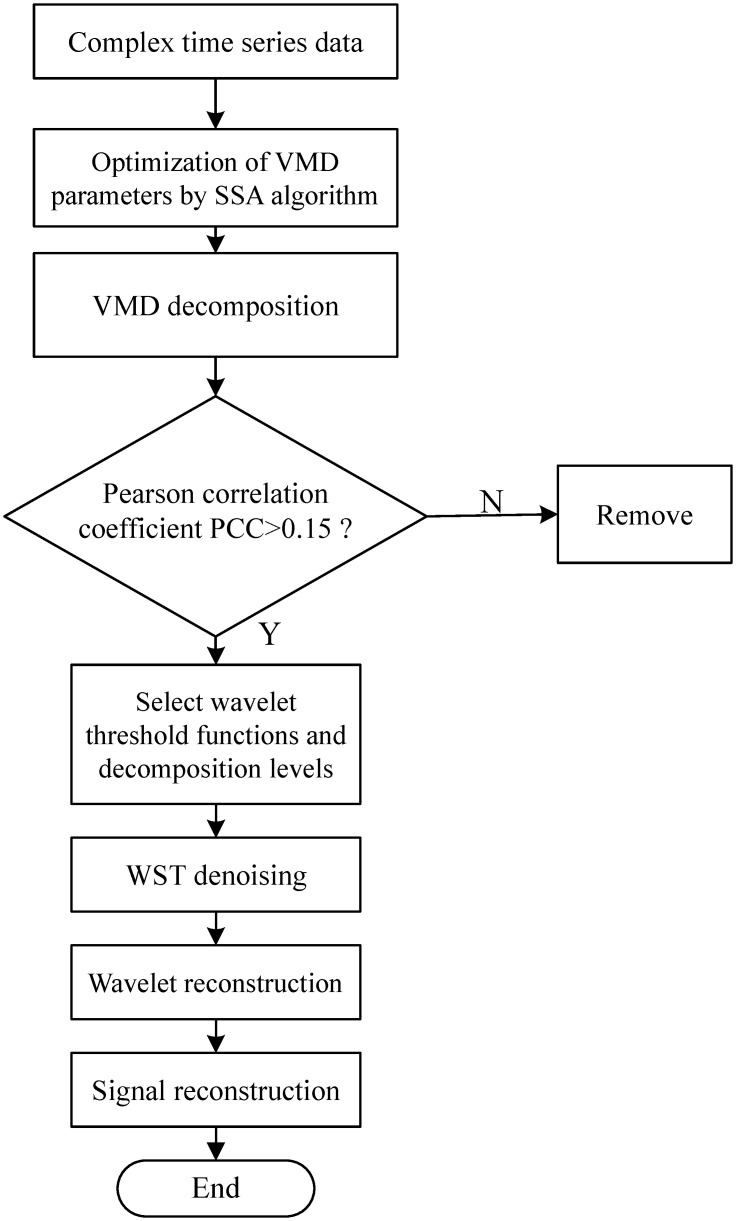
Flow of SSA-VMD-WST model.

In this paper, we optimize two parameters which can affect the VMD decomposition based on SSA algorithm, and then carry out WST denoising by analyzing the PCC of each component and the original sequence. The process is as follows in Fig 5: We optimize VMD using SSA algorithm to get the optimal parameter decomposition layer and penalty parameter. The optimized VMD is used to decompose the original signal. Then the PCC of the decomposed IMF component was calculated. The smaller PCC component was removed, and then the retained IMF component was denoised by wavelet threshold. The PCC definition of the IMF component is shown as Eq (26)

$$\begin{array}{c} r_{imf_{k},y}=\frac{cov(imf_{k},\sigma_{y})}{\sigma_{imf_{k}}\sigma_{y}} \end{array}$$ (26)

Reconstruct the signal to obtain the final signal.

## 4 Numerical experiment analysis

In order to verify the validity of proposed model, the daily maximum temperature data (Data from the http://data.cma.cn/data/cdcdetail/dataCode/A.0019.0001.S002.html) during 2019–2022 were selected as the samples in this paper.

### 4.1 VMD parameter optimization and signal filtering analysis

Bring in the daily maximum temperature data and use the SSA algorithm to optimize the parameters of VMD. Make the following settings before the experiment, the population number is set to 20, the maximum number of iterations is set to 15. The minimum *Fit* value is equal to 0.11569 after two iterations, the output decomposition layer *K* = 13 and quadratic penalty coefficient *α* = 1549.

**Fig 6:**
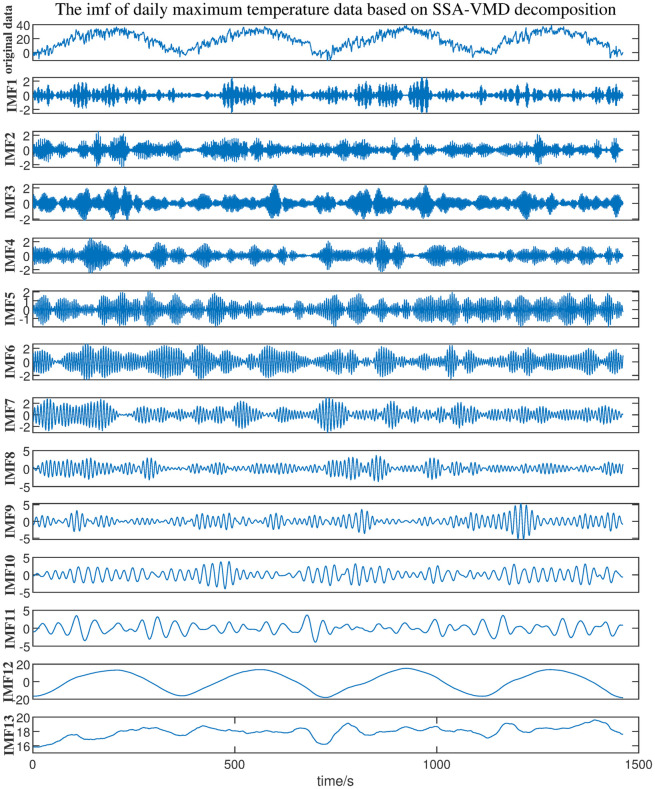
The 13 IMF components obtained by SSA-VMD decomposition of the original signal.

From Table 1, based on the root mean-square error(RMSE) and the mean absolute error(MAE) [36], the data error of decomposition parameters *K* = 13, *α* = 1549 are the smallest, MAE and RMSE are 0.326 and 0.416. Under the same *α*, as *K* value increases to 15, the data error of before and after denoising increased by 53.16% and 53.83%. So it can be considered that the signal has been completely decomposed when *K* = 13. Parameters *K* and *α* optimized by the SSA algorithm were input into Matlab, and 13 IMF components were shown as in Fig 6 after SSA-VMD decomposition.

**Table 1:** Data decomposition error under different parameters.

Argument	RMSE	MAE
*K* = 13, *α* = 1549	0.416	0.326
*K* = 13, *α* = 1049	0.569	0.359
*K* = 13, *α* = 2249	0.605	0.375
*K* = 12, *α* = 1549	0.689	0.402
*K* = 11, *α* = 1549	0.608	0.446
*K* = 15, *α* = 1549	0.901	0.696

In this paper, the threshold of PCC is set to 0.15. It is believed that when the PCC of IMF is greater than 0.15, we consider it is an effective mode. The retained IMF can express the features of the original signal with less information loss. The PCC of each IMF component are calculated shown in Table 2. It can be found that the PCC of IMF1∼IMF8 are all less than 0.15, and the PCC of reserved IMF9∼IMF13 are all greater than 0.15. It can be considered that IMF9∼IMF13 can preserve the signal characteristics greatly.

**Table 2:** Correlation between the IMF component and the original signal.

Index	Component	Correlation
PCC	IMF1	0.0695
	IMF2	0.0702
	IMF3	0.0836
	IMF4	0.0871
	IMF5	0.0999
	IMF6	0.1187
	IMF7	0.1204
	IMF8	0.1307
PCC	IMF9	0.1515
	IMF10	0.1589
	IMF11	0.1643
	IMF12	0.9396
	IMF13	0.3435

### 4.2 Analysis of denoising effect

**Fig 7:**
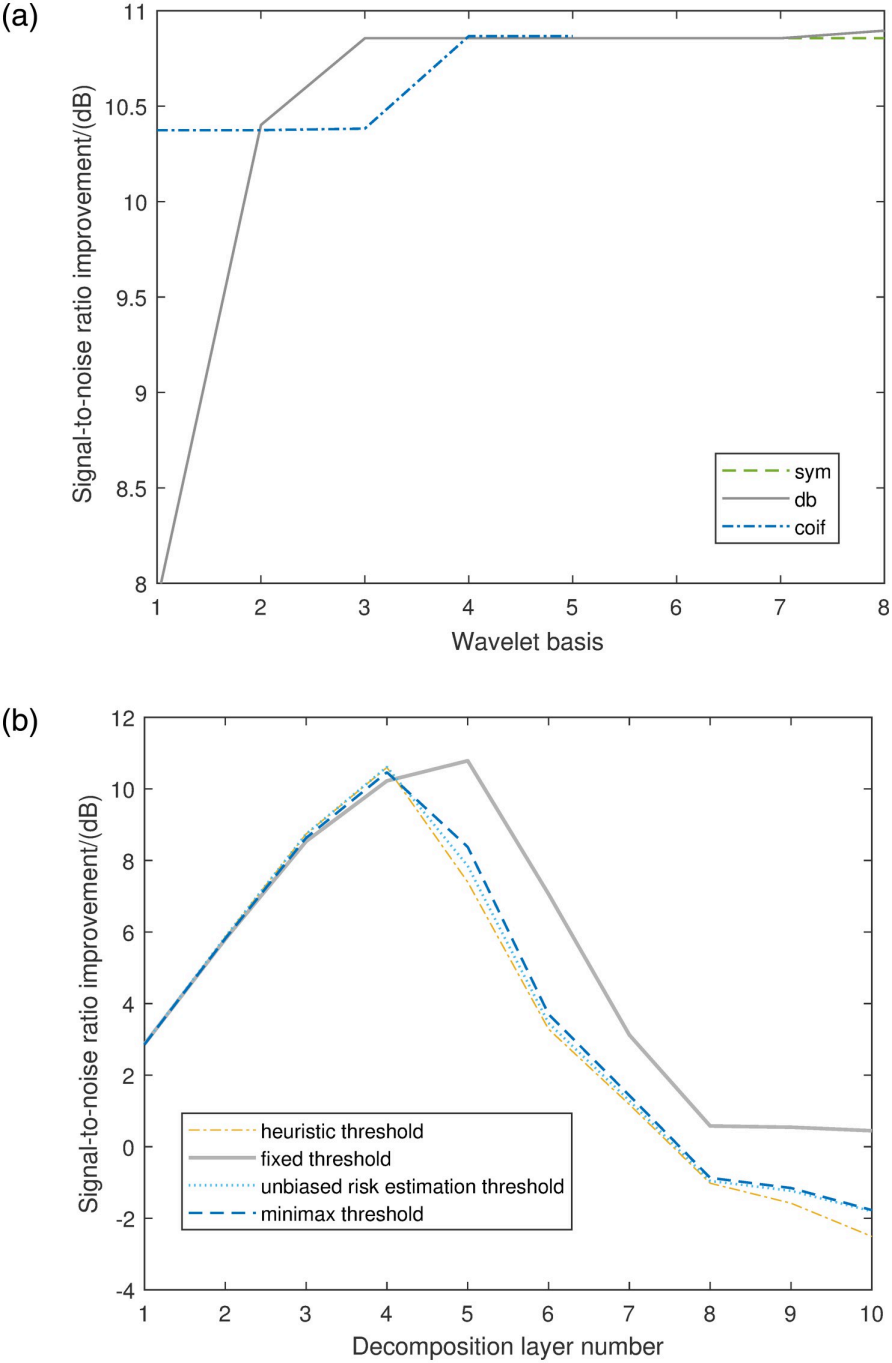
SNR under different wavelet bases, wavelet thresholds and decomposition layers.

**Fig 8:**
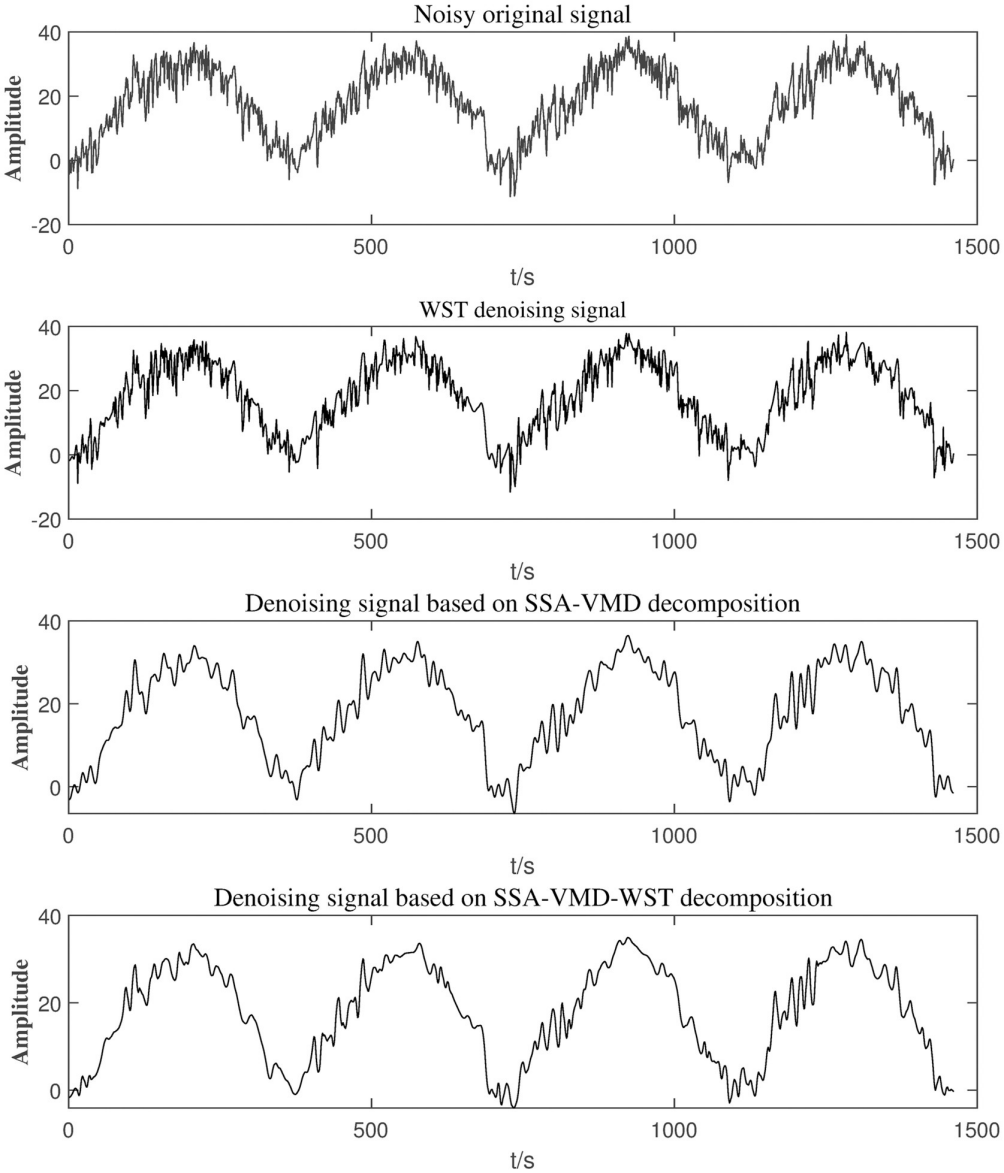
Data fluctuations under different denoising methods.

By substituting the data into the program, it is obtained that the most suitable wavelet threshold is fixed threshold, and the number of decomposition layers is 5 layers. At the same time, the best wavelet basis is db3. The results are shown in Fig 7. The comparison results between the original signal and the other denoising signal results are shown in Fig 8.

Compared with the above denoising signal diagram in Fig 8, the effect of SSA-VMD-WST denoising is smoother than SSA-VMD and WST methods. This shows that only after SSA-VMD denoising, noise is still left. In order to further evaluate the advantages of the SSA-VMD-WST, the signal denoising is quantitatively analyzed by means of four common signal index, MAE, RMSE [36], cross-correlation(CC) [37] and signal-to-noise ratio(SNR) [38].

The index comprehensively evaluate the effect of signal denoising. SNR refers to the ratio of useful signal to noise. Therefore, the higher SNR and CC of the signal after denoising, the smaller RMSE and MAE with original signal, the better denoising effect is. The evaluation index values of the three denoising methods are shown in Table 3.

**Table 3:** Comparison of index under different denoising methods.

Model	SNR	RMSE	MAE	CC
*WST*	17.1511	1.9573	1.5732	0.8997
*SSA* − *VMD*	17.9003	1.7129	1.2806	0.9002
*SSA* − *VMD* − *WST*	20.2383	1.0727	0.6143	0.9342

In Table 3, the RMSE, CC and SNR of the SSA-VMD-WST denoising method are 1.0727, 0.9342 and 20.2383, respectively, which are the best model. The SNR is the largest which indicates that the SSA-VMD-WST can retain the original signal information better than other methods and remove more noise than SSA-VMD and WST methods, and the CC is the largest which shows that the SSA-VMD-WST can ensure consistent data fluctuation before and after denoising.

### 4.3 Prediction of daily maximum temperature based on CNN-BiLSTM

**Fig 9:**
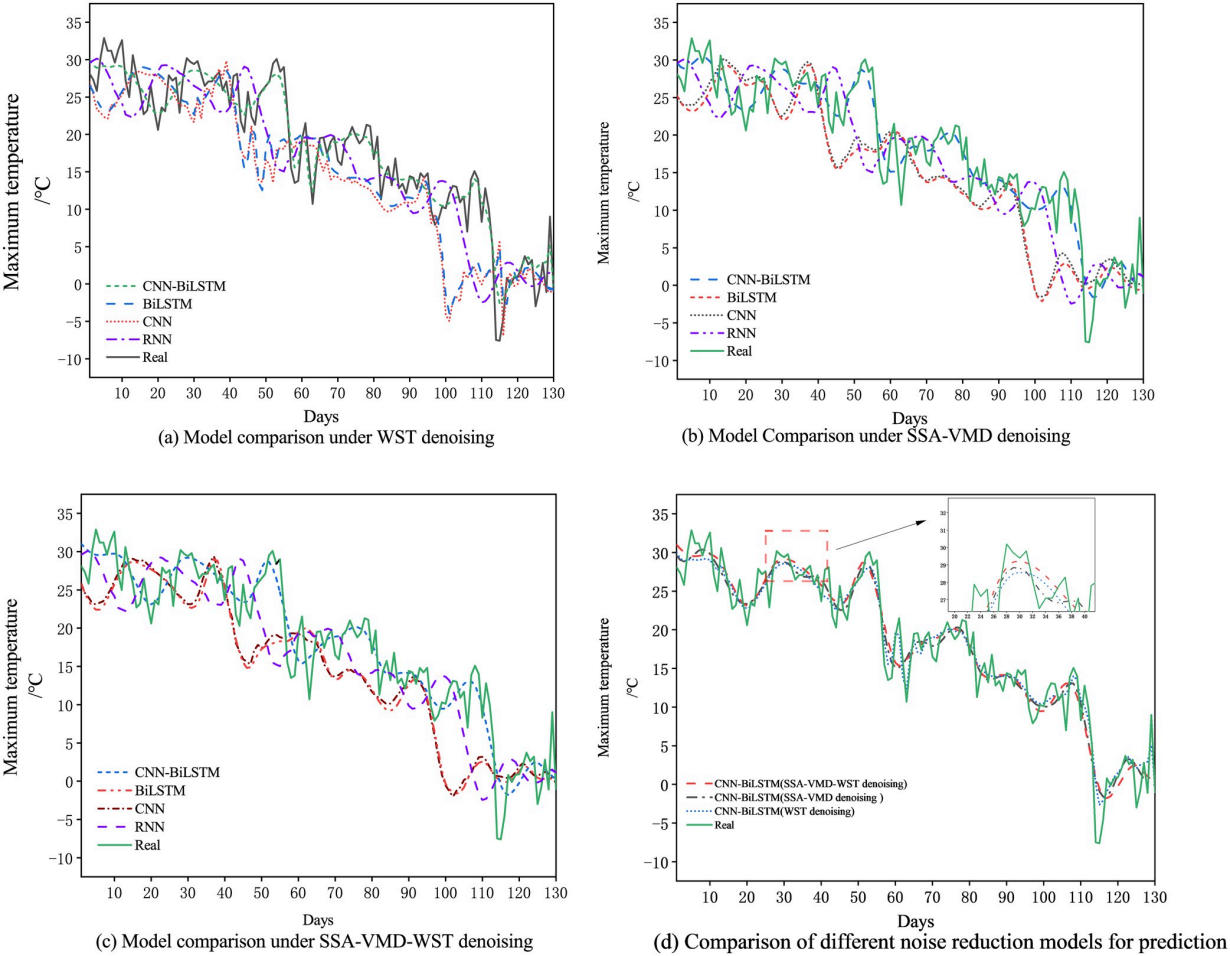
CNN-BiLSTM trend fitting of maximum temperature under different denoising methods.

Aiming at accuracy and rationality of the CNN-BiLSTM, the daily maximum temperature prediction is take an example to shown in Fig 9 and Table 4 compared with different denosing methods and different deep leaning methods.

**Table 4:** Comparison of prediction results.

Method	Model	RMSE	MAE	*R* ^2^
original data	RNN	2.159	1.743	0.8504
	CNN	4.005	3.350	0.8575
	BiLSTM	3.169	2.394	0.8593
	CNN-BiLSTM	1.389	0.939	0.8760
WST	RNN	1.034	0.906	0.8832
	CNN	1.382	0.890	0.8854
	BiLSTM	1.434	0.863	0.8866
	CNN-BiLSTM	0.683	0.488	0.8904
SSA-VMD	RNN	0.790	0.617	0.8924
	CNN	0.705	0.608	0.9014
	BiLSTM	0.556	0.336	0.9084
	CNN-BiLSTM	0.416	0.322	0.9088
SSA-VMD-WST	RNN	0.611	0.486	0.9167
	CNN	0.416	0.326	0.9207
	BiLSTM	0.507	0.408	0.9236
	CNN-BiLSTM	0.188	0.150	0.9364

After WST denosing, the daily maximum temperature predictions with real data, RNN, CNN, BiLSTM and CNN-BiLSTM are shown in Fig 9(a). From the figure we can find the CNN-BiLSTM with WST denosing has better fitting effect than RNN, CNN and BiLSTM with WST denosing. In Fig 9(b), we can know that there is better fitting effect for the CNN-BiLSTM with SSA-VMD denosing than RNN, CNN and BiLSTM. After SSA-VMD-WST denosing, the daily maximum temperature predictions with real data, RNN, CNN, BiLSTM and CNN-BiLSTM are shown in Fig 9(c). From the figure we can find the CNN-BiLSTM with SSA-VMD-WST denosing has better trend evolution. In Fig 9(d), we can know that there is better fitting effect for CNN-BiLSTM with real data for the SSA-VMD-WST compared with other denosing methods.

The trend fitting of the SSA-VMD-WST based on CNN-BiLSTM in Fig 9 is smoother and more accurate. At the same time, according to the data in the Table 4, the hybrid strategy which is combined SSA-VMD-WST with CNN-BiLSTM has the best prediction effect, RMSE and MAE are the smallest which are 0.188 and 0.150, the goodness of fit *R*^2^ is the highest, and its value is 0.9364. Compared with CNN-BiLSTM-WST error decreased by 72.47% and 84.03%. Meanwhile, in relation to CNN-BiLSTM-SSA-VMD error decreased by 54.80% and 53.42%. In Table 4, we also find that the data without noise reduction has the worst prediction effect, indicating that the data itself has a greater noise interfere.

## 5 Conclusions

With the aim to overcome the strong noise problem of complex time series data, a new hybrid denoising model SSA-VMD-WST is proposed based on the traditional complex time series denoising. Firstly, a new fitness function is constructed by using sample entropy and PCC, which is embedded into SSA to realize the comprehensive optimization of VMD uncertain parameters and establish SSA-VMD model. Then, VMD is applied to decompose and denoise the complex time series data. Considering the effectiveness of each subsequence, PCC method is used to filter the subsequence. The SSA-VMD-WST model is established after denoising the reserved subsequences by WST method. Finally, CNN-BiLSTM model is used to fit the trend of the denoised data. Compared with CNN-BiLSTM-SSA-VMD and CNN-BiLSTM-WST, the RMSE of CNN-BiLSTM-SSA-VMD-WST decreased by 54.80% and 72.47% respectively, and the MAE decreased by 53.42% and 84.03%, which indicates that the proposed method is suitable for nonlinear and nonstationary complex time series data denoising and prediction. In addition, some outliers may be removed as noise in the above process. Therefore, the prediction accuracy can be improved by further processing in the future research.

## Supporting information

S1 Data (ZIP)
